# Both Open and Arthroscopic Latarjet Result in Excellent Outcomes and Low Recurrence Rates for Anterior Shoulder Instability

**DOI:** 10.1016/j.asmr.2021.09.017

**Published:** 2021-11-04

**Authors:** Eoghan T. Hurley, Erel Ben Ari, Nathan A. Lorentz, Edward S. Mojica, Christopher A. Colasanti, Bogdan A. Matache, Laith M. Jazrawi, Mandeep Virk, Robert J. Meislin

**Affiliations:** NYU Langone Health, Orthopaedic Surgery Department, New York, New York, U.S.A.

## Abstract

**Purpose:**

The purpose of this study is to evaluate the patient-reported outcomes of open Latarjet (OL) compared to arthroscopic Latarjet (AL) for anterior shoulder instability.

**Methods:**

A retrospective review of patients who underwent either OL or AL for anterior shoulder instability between 2011 and 2019 was performed. Recurrent instability, visual analog scale (VAS) score, Shoulder Instability-Return to Sport after Injury (SIRSI), Subjective Shoulder Value (SSV), Western Ontario Shoulder Instability (WOSI) score, patient satisfaction, willingness to undergo surgery again, and return to work/sport (RTW/RTS) were evaluated. A *P* value of < .05 was considered to be statistically significant.

**Results:**

Our study included 102 patients in total; 72 patients treated with OL, and 30 treated with AL. There were no demographic differences between the two groups (*P* > .05 for all). At final follow up (mean of 51.3 months), there was no difference between those that underwent OL or AL in the reported WOSI, VAS, VAS during sports, SSV, and SIRSI scores, nor in patient satisfaction, or whether they would undergo surgery again (*P* > .05). Overall, there was no significant difference in the total rate of RTP (65% vs 60.9%; *P* = .74), or timing of RTP (8.1 months vs 7 months; *P* = .35). Additionally, there was no significant difference in the total rate of RTW (93.5% vs 95.5%; *P* = .75). Overall, 3 patients in the OL group and 2 patients in the AL group had recurrent instability events (6.9% vs 6.7%; *P* = .96), with no significant difference in the rate of recurrent dislocation (4.2% vs 3.3%; *P* = .84).

**Conclusion:**

In patients with anterior shoulder instability, both the OL and AL are reliable treatment options, with a low rate of recurrent instability, and similar patient-reported outcomes.

## Introduction

Anterior shoulder instability is a challenging clinical pathology to treat, and the Latarjet procedure is indicated in those with risk factors for postoperative recurrence, including those with severe glenoid bone loss, and those with a prior failed soft-tissue stabilization.^1^ The Latarjet procedure has been shown to result in lower rates of recurrent instability compared to Bankart repair, with studies showing a less than 5% recurrence rate at 10-year follow-up, and high rates of return to premorbid function and activity.^2^^,^^3^^,^^4^ Traditionally, the Latarjet procedure has been performed with an open approach (OL); however, there has been increased interest in the arthroscopic technique described by Lafosse et al.^5^

The arthroscopic Latarjet (AL) procedure has gained popularity due to its minimally invasive approach, which potentially results in decreased stiffness, fewer wound complications, and a quicker rehabilitation.^5^, ^6^, ^7^ Hurley et al. found in a meta-analysis that both the open and arthroscopic approach resulted in similar clinical outcomes, with no difference in recurrence rates. However, they did show there were lower immediate postoperative pain scores with AL. Driven by the potential benefits of performing this procedure arthroscopically, our senior author was an early North American adopter of this procedure.^8^ Thus, we sought to compare the clinical outcomes following arthroscopic Latarjet procedures to our institution’s experience with performing the open Latarjet procedure. Our group previously evaluated the 90-day complication rates of these two procedures, and we found no difference but did not have sufficient follow-up to compare clinical outcomes.^9^

The purpose of this study is to evaluate the patient-reported outcomes of open Latarjet (OL) compared to arthroscopic Latarjet (AL) for anterior shoulder instability. Our hypothesis was that there would be no significant difference in recurrence rates or functional outcomes between the two procedures.

## Methods

### Patients

Our Institutional Review Board approved this retrospective comparative study. All patients treated with either an open or arthroscopic Latarjet procedure for a diagnosis of anterior shoulder instability with glenoid bone loss between January 2012 and October 2019 were identified, and the chart was reviewed for eligibility. The inclusion criteria of the current study was age >16 at the time of surgery, skeletal maturity, preoperative imaging consisting of magnetic resonance imaging (MRI) and a minimum follow-up of 12 months. One reviewer and current orthopaedic surgery resident (EH) completed the necessary chart review to limit bias. The open procedure was performed by several surgeons at the investigating institution, but the arthroscopic approach was used by only one surgeon. Exclusion criteria consisted of connective tissue disease.

### Surgical Technique

#### Open Latarjet

All patients underwent surgery in a beach chair position using a combination of general anesthesia and an interscalene regional nerve block. The surgical technique was standardized across all surgeons. In brief, an anterior incision was used to access the shoulder via a deltopectoral approach. The cephalic vein was mobilized laterally in all cases. The coracoid was exposed, the coracoacromial (CA) ligament was released 1 cm lateral to insertion, and the pectoralis minor was released subperiosteally. A medial-to-lateral osteotomy of the coracoid base was then started using an angled saw and completed with an osteotome. The backside of the conjoint tendon was then bluntly mobilized until it reached the level of the musculocutaneous nerve. The inferior surface of the coracoid was then decorticated and contoured, and two central drill holes in the graft were made. Next, the subscapularis tendon was divided with a horizontal split at the upper 2/3-lower 1/3 junction, and either a vertical or horizontal capsulotomy was performed, according to surgeon’s preference. The anterior glenoid neck was then debrided of all residual tissue and lightly decorticated. The coracoid graft was then delivered through the subscapularis split and fixed to the glenoid using two cannulated screws. Closure of the capsule to the CA ligament was performed whenever possible with 2-0 FiberWire suture (Arthrex, Naples, FL).

#### Arthroscopic Latarjet

The arthroscopic Latarjet was also performed under general anesthesia and an interscalene nerve block while the patient was in the beach chair position with the addition of an arm positioner (Spider2, Smith & Nephew, Andover, MA). The surgical technique follows the steps outlined by Lafosse et al., modified by the senior surgeon. Six portals are used for this technique, which, in summary, consists of the following surgical steps: 1) preparation of the anterior glenoid neck; 2) rotator interval release; 3) anterior, superior, and posterior subscapularis release; 4) exposure of the coracoid and conjoint tendon; 5) subdeltoid bursoscopic CA ligament and pectoralis minor release; 6) coracoid osteotomy and graft preparation; 7) subscapularis split; 8) graft fixation using two 3.5-mm partially threaded, cannulated, cancellous screws.

### Data Collection and Clinical Outcomes

Data on patient characteristics and preoperative demographics were collected, including age, gender, laterality, glenoid bone loss, Hill-Sachs defect, and previous shoulder surgeries. Intraoperative and postoperative complications were recorded. Preoperative imaging was evaluated, and the degree of glenoid bone loss and extent of the Hill-Sachs lesion were evaluated by a musculoskeletal fellowship-trained radiologist. Evaluation of postoperative patient-reported outcomes was carried out following telephone survey, including visual analog scale (VAS) score, Subjective Shoulder Value (SSV), Western Ontario Stability Index (WOSI) score, satisfaction, and whether they would undergo the same surgery again. Additionally, the rate and timing of RTP and RTW and Shoulder Instability-Return to Sport after Injury (SIRSI) score were evaluated. Finally, recurrent instability (including dislocations and subluxations) was recorded.

### Statistical Analysis

Post hoc power analysis was performed for isolating differences in WOSI, and found that this study was powered at 95%. All statistical analysis was performed using GraphPad Prism 8.3 (GraphPad, La Jolla, CA). For all continuous and categorical variables, descriptive statistics were calculated. Continuous variables were reported as weighted mean and estimated standard deviation, whereas categorical variables were reported as frequencies with percentages. Categorical variables were analyzed using Fisher’s exact or χ^2^ test. The independent or paired *t*-test for normally distributed variables, or the nonparametric Mann-Whitney *U*-test or Wilcoxon signed-rank test was performed to compare continuous variables. A value of *P* < .05 was considered to be statistically significant.

## Results

### Patient Demographics

Overall, there were 110 patients treated with OL and 40 patients treated with AL. Of the 110 OL patients, 72 were available for follow-up, and of the 40 AL patients, 30 were available for follow-up. There were no significant differences in demographic variables between the groups. A comparison of patient demographics between OL and AL groups are further illustrated in Table 1**.**

**Table 1:** Patient Characteristics

	OL	AL	*P* Value
*n*	72	30	
Age	30 ± 10.5	32 ± 12.3	.29
Gender	32 (69.6%)	25 (83.3%)	.28
Glenoid bone loss	18% ± 7.6	18.6% ± 4.7	.90
Engaging Hill-Sachs	46 (100%)	30 (100%)	>.99
Prior surgery	36 (50%)	14 (46.7%)	.85
Follow-up (mo.)	53.9 ± 27	46.2 ± 26	.22

AL, arthroscopic Latarjet; mo, months; *n*, number; OL, open Latarjet.

### Functional Outcomes

At final follow-up at an average of 51.3 months, we found no difference between those that underwent OL or AL in the reported WOSI score (*P* = .43) or any of its components, VAS score (*P* = .40), VAS during sports (*P* = .51), SSV (*P* = .6062), SIRSI score (*P* = .85), satisfaction (*P* = .50), or whether they would undergo surgery again (*P* = .30). A comparison of patient-reported outcome groups is shown in Table 2.

**Table 2:** Functional Outcomes

	OL	AL	*P* Value
WOSI (%)	24.9% ± 26.9%	27.1% ± 25.7%	.70
WOSI physical (%)	22.6% ± 24.1%	26.5% ± 25.2%	.46
WOSI sport (%)	24.7% ± 28.8%	26.2% ± 27.6	.81
WOSI lifestyle (%)	23.5% ± 27.1%	21.8% ± 24.4	.77
WOSI emotional (%)	29.1% ± 29.1%	32.5% ± 32.2	.60
VAS	1 ± 2.2	1.3 ± 2	.52
VAS Sport	1.5 ± 2.2	2.2 ± 2.7	.17
SSV	74.1 ± 24.2	75.7 ± 22.1	.76
SIRSI	67 ± 24.1	66.7 ± 25.6	.96
Satisfaction	88.3% ± 17%	85.6% ± 17.7%	.47
Repeat surgery	69/72 (95.8%)	28/30 (93.3%)	.63

AL, arthroscopic Latarjet; OL, open Latarjet; SIRSI, shoulder instability-return to sport after injury; SSV, subjective shoulder value; VAS, visual analog scale; WOSI, Western Ontario Shoulder Instability score.

### Return to Play and Work

Overall, there was no significant difference in the total rate of RTP (*P* = .74), or timing of RTP (*P* = .35). Additionally, there was no significant difference in the total rate of RTW (*P* = .75). A comparison of RTP/RTW between the groups is shown in Table 3.

**Table 3:** Return to Play/Work

	OL	AL	*P* Value
RTP	26/40 (65%)	14/23 (60.9%)	.74
RTP timing (mo.)	8.1 ± 3.7	7 ± 3	.35
RTW	58/62 (93.5%)	21/22 (95.5%)	.75

AL, arthroscopic Latarjet; mo, months; OL, open Latarjet; RTP, return to play; RTW, return to work.

### Recurrent Instability

Overall, 5 patients in the OL group and 2 patients in the AL group had recurrent instability events (*P* =.96), with no significant difference in the rate of recurrent dislocation between the groups (*P* = .84). Four patients in the OL (5.6 %) patient required a revision to distal tibial allograft for recurrent instability, and no patients required a revision in the AL group. A comparison of recurrent instability between the OL and AL groups are illustrated in Table 4.

**Table 4:** Recurrent Instability

	OL	AL	*P* Value
Total recurrence	5 (6.9%)	2 (6.7%)	.96
Redislocations	3 (4.2%)	1 (3.3%)	.84
Subluxation	2 (2.7%)	1 (3.3%)	.88

AL, arthroscopic Latarjet; OL, open Latarjet.

### Complications

There was no significant difference in the overall complication rate between OL and AL (11.1% vs 6.7%; *P* = .72) or in the revision rate (8.3% vs 3.3%; *P* = .67).

## Discussion

The most important finding in our study was that both OL and AL result in excellent and similar clinical outcomes, with no difference in either functional outcomes or recurrence rates between the two approaches. Additionally, despite concerns that the arthroscopic approach may lead to higher complication rates and ultimately higher revision rates, there was no observed difference between open and arthroscopic approaches. The findings from this study further the body of evidence in the literature on the AL procedure, and support its equivalent standing with OL.

The Latarjet procedure has seen a resurgence in popularity in recent years, because of its high success rates, with studies reporting excellent long-term shoulder stability and low recurrence rates.^3^^,^^10^, ^11^, ^12^ Therefore, it has been advocated for in patients with high risk factors for recurrence after an arthroscopic Bankart repair, with several scoring systems available to stratify risk, primarily focusing on glenoid bone loss, Hill-Sachs lesions, and involvement in collision sports.^13^, ^14^, ^15^, ^16^ Because of Latarjet’s nonanatomical nature and invasive approach, there have been concerns over the associated risk of complications,^17^^,^^18^ such as bone block nonunion or resorption, fracture of the coracoid, or injury of the musculocutaneous nerve, either during the approach or during retraction.^17^^,^^18^ In an effort to reduce this, Lafosse et al. described an arthroscopic approach in 2007, and this has shown favorable outcomes at short-to-midterm follow-up.^5^ However, this is a technically challenging approach, and surgeons have cautioned against its use in low-volume centers, as there is a significant learning curve, even for experienced arthroscopists.^7^^,^^9^

Overall, our study found no differences in any outcome measure following the OL and AL procedures. The primary functional outcome measure used was the WOSI score. This has been validated to assess the impact of shoulder instability across a variety of lifestyle domains, all of which showed no significant difference between the two groups.^19^ At final follow-up, at an average of 51.34 months, there was no significant difference in pain levels; however, Hurley et al.^7^ found in their meta-analysis that in initial postoperative period that AL resulted in decreased pain levels compared to OL, which was the only functional outcome score in which they found any difference. Furthermore, there was no difference in the current study in SSV, satisfaction, or willingness to undergo surgery again.

The most important outcome for athletes following shoulder instability surgery is whether they are able to RTP postoperatively.^20^^,^^21^ There was no difference in overall rate of RTP between the two groups, and while the rate may seem lower than quoted in the literature, this may be due to a large portion of these patients having a prior surgery and the average age being in their 30s, both of which are risk factors for not returning to play in our experience.^4^ Although there was a small difference in timing of RTP by 1 month favoring AL, the potential for quicker rehabilitation following AL is an often discussed advantage and does warrant further study. The Latarjet procedure is often reported as allowing faster RTP than arthroscopic Bankart repairs, because bony healing is quicker than soft-tissue healing, and the AL approach may be further advantageous because of its minimally invasive approach.^4^^,^^22^ Finally, there was no difference in SIRSI scores between the two groups, which is a measure of psychological confidence in patients’ ability to return to play.^23^

The recurrence rates were similar in both techniques in terms of recurrence, suggesting both are equally efficacious in treating anterior shoulder instability. Both groups had the same fixation using cancellous screws, and thus, any potential differences would likely be present as a result of graft positioning, which may contribute to the steep learning curve of the AL.^24^^,^^25^ However, our study did not perform any postoperative radiological analysis of the graft and screw fixation as postoperative imaging was not routinely obtained on all patients. Kordasiewicz et al.^24^ evaluated the learning curve for graft positioning and found a threshold of 30 cases. Furthermore, they found in another study using CT analysis that the AL approach resulted in improved screw positioning, and more superior graft positioning than with the OL approach.^25^

Overall, there were no differences in complication or revision rates between the two groups in our study. In both groups, there was 1 revision to distal tibial allograft for graft fracture, which is one of the most common complications of the Latarjet procedure. Additionally, there were wound infections in both groups, but only one patient in the OL group required an irrigation and debridement. While this is a rare complication, this is one of the purported advantages of the arthroscopic technique, as it is minimally invasive and may have less associated soft tissue and wound complications.^5^ Infections in the shoulder area have the potential for significant morbidity, especially in the case of *Cutibacterium acnes*, which can grow insidiously, is difficult to treat, and may result in lingering pain due to subclinical infection.^26^

### Limitations

There are several potential limitations to our study. First, it is a retrospective study, and thus, it is subject to potential bias. Additionally, we are limited by the fact that the arthroscopic procedures were only performed by a single surgeon, whereas the open procedures were performed by multiple surgeons. However, we felt that the comparison is still appropriate, given the fact that most centers that perform the arthroscopic Latarjet do so via an “early adopter” or a limited few, while the remainder perform it open. Radiographic comparison was not done to determine the graft position obtained with arthroscopic versus open Latarjet.

### Conclusion

In patients with anterior shoulder instability, both the open and arthroscopic Latarjet procedures are reliable treatment options, with a low rate of recurrent instability, and similar patient reported outcomes.

## Supplementary Data

ICMJE author disclosure forms
